# Comparison between the disease-specific Airways Questionnaire 20 and the generic 15D instruments in COPD

**DOI:** 10.1186/1477-7525-9-4

**Published:** 2011-01-16

**Authors:** Witold Mazur, Henna Kupiainen, Janne Pitkäniemi, Maritta Kilpeläinen, Harri Sintonen, Ari Lindqvist, Vuokko L Kinnula, Tarja Laitinen

**Affiliations:** 1 Department of Medicine, Pulmonary Division, Helsinki University Central Hospital, Haartmaninkatu 4, 00029 Helsinki, P.O. Box 372, Finland; 2 Department of Public Health, University of Helsinki, Mannerheimintie 172, 00014 Helsinki, P.O. Box 41, Finland; 3 Department of Medicine, Pulmonary Division, Turku University Central Hospital, Kiinamyllykatu 4-8, 20520 Turku, Finland; 4 Department of Medicine, Pulmonary Division, Tampere University Central Hospital, Teiskontie 35, 33521 Tampere, Finland

## Abstract

**Background:**

Given that the assessment of health-related quality of life (HRQoL) is an essential outcome measure to optimize chronic obstructive pulmonary disease (COPD) patient management, there is a need for a short and fast, reliable and valid instrument for routine use in clinical practice. The objective of this study was to analyse the relationship between the disease-specific Airways questionnaire (AQ20) and the generic 15D health-related quality of life (HRQoL) instrument simultaneously in a large cohort of patients with COPD. We also compare the HRQoL of COPD patients with that of the general population.

**Methods:**

The AQ20 and 15D were administered to 739 COPD patients representing an unselected hospital-based COPD population. The completion rates and validity of, and correlations among the questions and dimension scores were examined. A factor analysis with varimax rotation was performed in order to find subsets of highly correlating items of the questionnaires.

**Results:**

The summary scores of AQ20 and 15D were highly correlated (r = - 0.71, p < 0.01). In AQ20 over 50% of patients reported frequent cough, breathlessness during domestic work, and chest problem limiting their full enjoyment of life. 15D results showed a noteworthy decrease of HRQoL in breathing, mobility, sleeping, usual activities, discomfort and symptoms, vitality, and sexual activity (scores ≤ 0.75). Compared to the age- and gender-standardized Finnish general population, the COPD patients were statistically significantly worse off on 13 of 15 dimensions.

**Conclusions:**

The AQ20 and 15D summary scores are comparable in terms of measuring HRQoL in COPD patients. The data support the validity of 15D to measure the quality of life in COPD. COPD compromises the HRQoL broadly, as reflected by the generic instrument. Both questionnaires are simple and short, and could easily be used in clinical practice with high completion rates.

## Introduction

Chronic obstructive pulmonary disease (COPD), a serious debilitating condition with worldwide prevalence of 8-20% today, is estimated to be the third leading cause of death by year 2020 [1-3]. Respiratory conditions in COPD such as emphysema or chronic bronchitis, or both, are related to (nearly) irreversible airway obstruction causing chronic cough or phlegm and breathlessness (dyspnea) [4]. Persistent and progressive dyspnea forces into lifestyle adjustments, impairs patients' health-related quality of life (HRQoL), and leads to disability. Since there is no medical or surgical cure for COPD with prognostic significance, one of the principal goals of the management of COPD is to improve patient's HRQoL by relieving symptoms and maintaining patient's physical and emotional capabilities [5].

HRQoL has become an established outcome measure that can be used to monitor and manage COPD. HRQoL can be evaluated by means of disease-specific or generic instruments. The disease-specific instruments focus on a particular condition and its effect on a patient's health [6,7]. Generic instruments are broad in their scope and applicability enabling the comparisons between different diseases and their treatments. Instead of concentrating on a single condition these questionnaires are designed to capture also the impact of co-morbidities and other quality of life impairing factors. Multiple profile questionnaires such as Short Form 36-item Questionnaire (SF-36), Sickness Impact Profile (SIP), Nottingham Health Profile (NHP), have been tested in COPD [8-15]. Multi-dimensional preference-based utility scales enable cost-utility analyses that are currently the most useful method in economic evaluation of health care interventions. The most commonly used generic utility instruments in pulmonary diseases are the EQ-5D, the Health Utility Index (HUI) and the 15D [16-19]. Given that the assessment of HRQoL is an essential outcome measure to optimise COPD patient management and to evaluate the effectiveness of therapeutic interventions, there is a need for a reliable and valid instrument for routine use in clinical practice.

The AQ20 and the 15D are two well-validated questionnaires that have been applied in the clinical assessments of HRQoL of patients suffering from obstructive pulmonary disorders such as asthma and COPD [6,7,20-27]. However, to our knowledge, there has been no comparative evaluation of these two questionnaires in COPD patients. In this study in a large cohort of patients with COPD we compare both these instruments and examine their applicability. We assess the convergent validity of the generic 15D using disease-specific AQ20 and examining the correlations among the items of both instruments. In addition we explore how and to what extent the HRQoL in patients with COPD as measured by 15D differs from that of the general population.

## Materials and methods

### Subjects

This study belongs to a large clinical study of a cohort of COPD patients in Finland [28]. Shortly, all patients with COPD who had visited the Pulmonary Clinics of the Helsinki and Turku University Hospitals during the years 1995-2006 were identified from the Hospital Discharge Registries. The databases were screened by ICD10 code J44.8 and contained all patients between 18 to 75 years of age. The inclusion criterion was a diagnosis of COPD based on post-bronchodilatation spirometry according to GOLD criteria [29]. The research visits occurred during the years 2005-2007. All participants (N = 844) gave their informed consent to allow the research consortium to collect, merge, and analyze their comprehensive medical history from all healthcare providers who had treated them during the past 5-10 years and agreed to continue their follow-up on an annual basis for the next 10 years [28].

The HRQoL of patients was compared with that of a sample of the general Finnish population. The 15D data for the general population came from the National Health 2000 Health Examination Survey representing the Finnish population aged 30 years and older [30]. For this analysis those individuals were selected, who were in the age range of the patients (N = 5604). This sample was weighted to reflect the age and gender distribution of the patients.

### Assessment of the HRQoL

The HRQoL was assessed using the self-completed airway-specific AQ20 [21] and the generic 15D [31] instrument. All participants filled in both questionnaires at the same time during the research visit. The 15D instrument is a generic, multidimensional, standardized, self-administered evaluative tool of HRQoL that can be used both as a single index measure, and as a profile measure [31]http://www.15d-instrument.net/15D. It describes the health status with 15 dimensions, namely: mobility, vision, hearing, breathing, sleeping, eating, speech, elimination, usual activities, mental function, discomfort and symptoms, depression, distress, vitality, and sexual activity. Each dimension comprises five answer options. A single index score (the 15D score, also referred to here as 15D summary score) is obtained by incorporating population-based preference weights to the dimensions [31]. The maximum score is 1 (no problems on any dimension) and the minimum score is 0 (being dead). More generally, in all important properties (reliability, validity, discriminatory power and responsiveness) the 15D compares at least equally with other preference-based generic HRQoL instruments such as the EQ-5D, SF-6D and HUI3 [10,31,32]. The reliability, validity and responsiveness of 15D questionnaire has been established in a group of 59 patients with moderate COPD [27]. The summary score correlated well with commonly used clinical measures of symptoms, lung function, and exercise capacity.

As the disease-specific instrument of HRQoL we used the AQ20 questionnaire. AQ20 was developed in 1998 for use in asthma [21,25] and COPD [9,20] and translated into Finnish [21,25]. The AQ20 is a uni-dimensional measure containing 20 items with "yes" responses scored as 1, and "no" and "not applicable" scored as 0. The scores of 1 are summed up to obtain the AQ20 summary score, which ranges from 0 to 20. Score 0 indicates no impairment. [6,7]. In terms of discriminative properties and responsiveness, the AQ20 was found comparable with more complex questionnaires such as St. George's Respiratory Questionnaire (SGRQ) [12] and Chronic Respiratory Disease Questionnaire (CRQ) [24,33]. In a recent COPD study, the reproducibility of AQ20 and its excellent correlation with SGRQ were further corroborated [20].

The Coordinating Ethics Committee of the Helsinki and Uusimaa Hospital District approved the study approach, and the permission to conduct this research was granted by the Helsinki and Turku University Hospitals. All recruitment processes were well documented, the study personnel trained, and monitored to meet the standards of good clinical practice.

### Statistical analysis

All analyses were performed by the statistical software packages SPSS (version 16.0; Chicago, IL, USA). The distribution of responses across the two instruments, specifically at the top and bottom of the scale, was examined to identify possible ceiling or floor effects. Factor analysis technique was used to reduce and rearrange the items of the two instruments and thus identified the factors of related variables e.g. the groups of questions that measured the related components of HRQL in each questionnaire separately. To compare the factors, the factor analysis with orthogonal varimax rotation was performed, and maximum likelihood was used as the extraction method. Factors with eigenvalues 1 or greater were considered significant. Spearman's correlation coefficient (R) was used to estimate the correlation between the original items and the factor scores of both instruments. A p < 0.05 was considered significant.

## Results

### Patient recruitment and selection

A total of 844 patients participated in the study. A detailed description of the cohort including a complete list of inclusion and exclusion criteria has been published elsewhere [28]. Briefly, the previously given COPD diagnosis was re-evaluated. This evaluation led to the exclusion of 105 patients. Thus, a final cohort of 739 eligible patients (mean age ± SD; 64 ± 6.8 years, N = 473 men) with COPD and smoking-related symptomatic chronic bronchitis was included in the analyses. Basic clinical characteristics for the 739 participants of the study are shown in the original publication [28].

### Evaluation of the HRQoL

**Figure 1:**
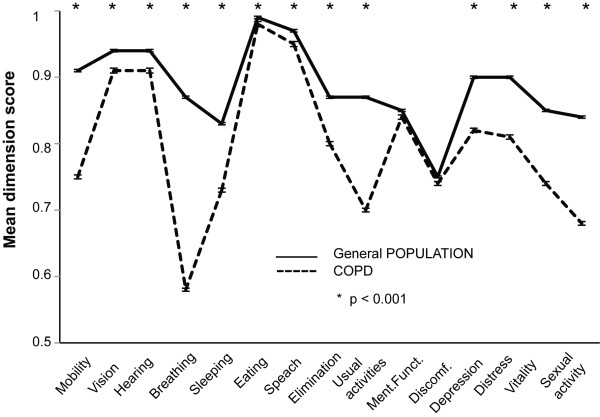
**The mean (SEM) values of the 15D dimensions in the patients with COPD disease and the control subjects from the general population**. * p < 0.001.

All the participants returned the HRQoL questionnaires. The proportions of ambiguous and missing responses per question in the HRQoL questionnaires were between 1-2%. Compared to the age- and gender-standardized Finnish general population, the COPD patients were statistically significantly worse off on all 15D dimensions except "mental function" and "discomfort and symptoms" (Figure 1). The mean 15D score of the COPD patients was 0.79 (± SD 0.11), which was significantly lower than that of the age- and gender-standardized general population (0.89 ± SD 0.09, p < 0.001). The 15D results showed a substantial decrease of HRQoL on several dimensions, especially in breathing, mobility, sleeping, usual activities, discomfort and symptoms, vitality, and sexual activity (scores ≤ 0.75) (Table 1). In the COPD patients the mean AQ20 summary score was 8.25 (± SD 5.0). In AQ20 more than 50% of the patients reported frequent cough, breathlessness during domestic work, and chest problem limiting the patient fully enjoy their life (Table 2). The AQ20 summary scores showed a small "ceiling effect": 33 patients (4.5%) did not present any respiratory symptoms and scored the best possible result (score 0). For the 15D, the highest possible score (= 1) was observed in 8 patients.

**Table 1:** Summary of the participants' responses to the questions in the 15D questionnaire.

Health Dimension	Level value Mean (SD)
Mobility	0.75 (0.17)

Vision	0.90 (0.17)

Hearing	0.91 (0.15)

Breathing	0.57 (0.22)

Sleeping	0.73 (0.23)

Eating	0.98 (0.08)

Speech	0.95 (0.12)

Elimination	0.80 (0.21)

Usual activities	0.7 (0.24)

Mental function	0.85 (0.19)

Discomfort and symptoms	0.74 (0.22)

Depression	0.83 (0.18)

Distress	0.81 (0.19)

Vitality	0.73 (0.18)

Sexual activity	0.67 (0.30)

**Table 2:** Summary of the participants' responses to the questions in the AQ20 questionnaire.

Number	Question	Number of subjects answered "Yes" (%)
AQ 1	Do you suffer from coughing attacks during the day?	403 (55)

AQ 2	Because of your chest trouble do you often feel restless?	311 (42)

AQ 3	Because of your chest trouble do you feel breathless maintaining the garden?	466 (64)

AQ 4	Do you worry when going to a friend's house that there might be something there that will set off an attack of chest trouble?	114 (15)

AQ 5	Do you suffer from chest symptoms as a result of exposure to strong smells, cigarette smoke or perfume?	361 (49)

AQ 6	Is your partner bothered by your chest trouble?	228 (31)

AQ 7	Do you feel breathless while trying to sleep?	213 (29)

AQ 8	Do you worry about the long term effects on your health of the drugs that you have to take because of your chest trouble?	232 (32)

AQ 9	Does getting emotionally upset make your chest trouble worse?	352 (48)

AQ 10	Because of your chest trouble are there times when you have difficulty getting around the house?	161 (22)

AQ 11	Because of your chest trouble do you suffer from breathlessness carrying out activities at work?	349 (48)

AQ 12	Do you feel breathless walking upstairs because of your chest trouble?	630 (85)

AQ 13	Because of your chest trouble do you suffer from breathlessness doing housework?	342 (46)

AQ 14	Because of your chest trouble do you go home sooner than others after a night out?	116 (16)

AQ 15	Because of your chest trouble do you suffer from breathlessness when you laugh?	97 (13)

AQ 16	Because of your chest trouble do you often feel impatient?	217 (30)

AQ 17	Because of your chest trouble do you feel that you cannot enjoy a full life?	443 (60)

AQ 18	Do you feel drained after a cold because of your chest trouble?	396 (54)

AQ 19	Do you have a feeling of chest heaviness?	351 (47)

AQ 20	Do you bother much about your chest trouble?	316 (43)

### Correlations between the 15D with AQ20 questionnaires in COPD patients

**Figure 2:**
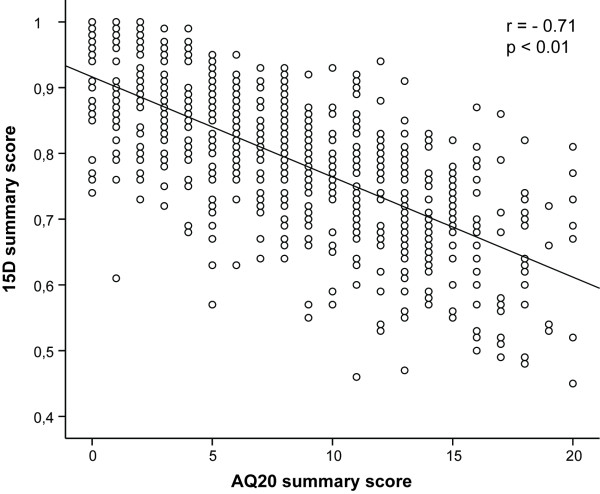
**15D and AQ20 summary scores were significantly correlated**.

The 15D and AQ20 summary scores were highly correlated (R = - 0.71, p < 0.01) (Figure 2). Due to the opposite scales, all correlations were negative. The factor analysis yielded 4 factors for both questionnaires accounting for 42% of the total variance in 15D and 38% in AQ20 (Table 3). The identified factors were partially similar. The most important factor in both questionnaires identified was 'Limitations in physical activity' (16% and 13% of variance, respectively). Both questionnaires also identified an emotional dimension among the patients. 'Psychic wellbeing' explained 15% of variance in 15D and 'Emotional concern' 9% in AQ20. AQ20 found also factors for symptoms at rest and during daily/social activities, factors that were missing from 15D. In order to estimate the degree to which the factors are inter-correlated, the resultant four factors were compared in a simple correlation matrix. The factor 'Limitations in physical activity' in 15D correlated significantly with the factors 'Limitations in physical activity' (R = - 0.65, p < 0.0001), 'Symptoms at rest' (R = - 0.22, p < 0.0001), and 'Emotional concern' (R = - 0.31, p < 0.0001), and 'Limitations in daily activities' (R = - 0.19, p < 0.0001) in AQ20. 'Psychic wellbeing' in 15D correlated significantly with 'Symptoms at rest' (R = - 0.30, p < 0.0001) and 'Emotional concern' in AQ20 (R = - 0.34, p < 0.0001). 'Capability of thinking and speaking' in 15D correlated with 'Symptoms at rest' (R = - 0.22, p < 0.0001) and 'Emotional concern' of AQ20 (R = - 0.16, p < 0.0001), and 'Limitations in daily activities' (R = - 0.13, p < 0.002). 'Eating' in 15D correlated with 'Limitation in physical activity' (R = - 0.09, p < 0.02), and 'Symptoms at rest' (R = - 0.15, p < 0.0001) in AQ20. The individual questions of AQ20 correlated strongly (R from -0.40 to - 0.54) with the 15D dimensions of mobility (AQ20 items 3, 10, 12, 13), breathing (AQ20 items 3, 10, 11, 12, 13, 17, 20), usual activities (AQ20 items 3, 11, 13, 17, 20), and vitality (AQ20 items 3, 11, 13, 17, 20). There existed a significant correlation between AQ20 summary score and sexual functioning in the whole cohort (R = - 0.48, p = 0.01). The lowest or not significant correlations were observed for vision, hearing, eating, elimination and speech (data not shown).

**Table 3:** Rotated factor loadings of the factors of the original items of the 15D questionnaires and the AQ20 questionnaire.

15D questionnaire			AQ20 questionnaire		
**Name of the factor**	**Original item**	**Rotated factor loadings**	**Name of the factor**	**Original item**	**Rotated factor loadings**

**"Limitation in physical activity" **(15.7% of the variation)	Usual activities Breathing Mobility Vitality Sexual activity	0.77 0.75 0.66 0.55 0.47	**"Limitation in physical activity" **(13.3% of the variation)	AQ3 AQ13 AQ11 AQ12 AQ17	0.76 0.70 0.64 0.49 0.48

**"Psychic wellbeing" **(14.7% of the variation)	Depression Distress Vitality	0.89 0.79 0.54	**"Symptoms at rest" **(10.2% of the variation)	AQ5 AQ18 AQ9 AQ19 AQ7	0.56 0.48 0.47 0.44 0.42

**"Capability of thinking and speaking" **(8.1% of the variation)	Mental functions Speech	0.55 0.42	**"Emotional concern" **(8.9% of the variation)	AQ2 AQ16 AQ20 AQ6 AQ17	0.58 0.55 0.54 0.45 0.44

**Eating **(3.9% of the variation)	Eating	0.40	**"Limitations in daily activities" **(6.0% of the variation)	AQ14 AQ10 AQ15	0.57 0.43 0.41

All factors with eigenvalues more >1 and factor loading ≥0.4 were included, maximum likelihood was chosen as the extraction method. The rotational method used was orthogonal, e.g. Varimax.

## Discussion

To our knowledge, the present report is the first study to evaluate the HRQoL in a large unselected hospital-based population of patients with stable COPD using the 15D as a generic HRQoL measure and the AQ20 as a disease-specific HRQoL measure at the same time. COPD compromises the HRQoL widely, as reflected by the generic instrument 15D, which demonstrated a clearly poorer quality of life in the patients compared with the general population sample in 13 of the 15 dimensions evaluated. Scores of the corresponding questions and dimensions of AQ20 and 15D, respectively, and the summary scores of both instruments correlated significantly, supporting the convergent validity of 15D to evaluate quality of life in COPD. The study showed that both the questionnaires comprise common elements, but also instrument-specific features. Furthermore, both questionnaires are simple and short, and could easily be used in clinical practice with high response and completion rates.

Contrary to conventional respiratory HRQoL measures AQ20 is less comprehensive, although fully applicable to COPD [22,24]. The great advantage of the questionnaire is that it includes only 20 questions, which are easy and quick for the patient to fill in (4 minutes) and for the researcher to score (8 seconds). A small 'ceiling effect' was observed in the study population, most likely due to the simple design of the 20 items with 0/1 responses only and non-identification of mild symptoms leading to avoidance of certain physical activities [24]. In order to omit this potential limitation, Chen and colleagues examined a modified version of AQ20, but did not observe a significant impact in its performance [23]. In this unselected hospital based COPD cohort, the mean summary score of AQ20 is consistent with the scores reported previously in COPD (8.33 vs. 5.9 - 9.9, respectively) [20,23,24]. However, in previous studies the sample sizes have been small (less than 200 study subjects) and gender distribution biased significantly towards males. Since our study design was cross sectional we were unfortunately not able to assess the evaluative properties of the AQ20 such as responsiveness.

The most substantial advantage of the generic preference-based utility HRQoL instruments is that they allow comparisons across diseases and cost-utility or cost-effectiveness analyses in health care. The main concern is whether these instruments are explicit and sensitive enough in specific diseases, especially at their mild and moderate disease's stages. In COPD few preference instruments, such as the EQ-5D, the Quality of Well Being Scale (QWB), or the HUI have been used [13,14,17,19,34-37], some of them with ambiguous results. A descriptive section of the EQ-5D has shown substantial ceiling effect [19,38]. One reason for this could be a three level classification of the health problem in comparison to 15D which allows the patient to express the problem on five levels. The ceiling effect is better avoided in the second part of the EQ-5D when visual analog scale (VAS) is used as a "health thermometer". In VAS patients' health status is evaluated on a continuous scale between the worst and the best imaginable health (scale 0-100). VAS has performed well in COPD studies and provided reliable and valid scores [19,39]. Direct comparisons of multiple health status instruments suggest that QWB may be less responsive in detecting health changes in patients who have undergone pulmonary rehabilitation than some of the disease-specific measures [40,41]. The 15D instrument has been previously used in COPD twice with different objectives. Compared to EQ-5D 15D was found more attractive due to better reliability and responsiveness in moderate COPD when co-morbidities were excluded [27]. In a large Finnish population survey (N = 6681), a total of 29 chronic conditions were studied using 15D and EQ-5D simultaneously [26]. Although formal comparisons of the two HRQoL measures were not performed, this study found significant and systematic differences in the rank order of disease severity between the two measures. 15D appeared to emphasize the relative impact of lung diseases while EQ-5D ranked COPD as less severe. Furthermore, like in most of the conditions studied, the HRQoL loss (standardised for a number of variables) was greater measured by the EQ-5D than by the 15D, but the former presented a much higher ceiling effect (25% vs. 5%, respectively). In the present study the mean HRQoL was lower compared to that in the survey (mean 15D score 0.79 vs. 0.84) most probably due older mean age and hospital-based recruitment biasing the cohort potentially towards more severe cases.

By definition the airway-specific and generic instruments focus on a single or multiple conditions, respectively, related to individual's HRQoL. In this study in the unselected population of COPD patients we found that the two HRQoL scales are highly correlated (assuming from the summary scores). These correlation data lend support for the assertion that the two instruments measure something similar. The factor analysis suggests that the instruments measure different aspects of the same concept, namely the overall effect of COPD on an individual's physical, psychical and emotional health. The first factor, generated from the two instruments, applies to the key problem caused by COPD, i.e. the limitation in physical activity relating to varying degrees in shortness of breath. Patients with COPD in general have a higher prevalence of depression and anxiety [42], and evidence suggests that these mental disorders account for a significant amount of variance in HRQoL, above and beyond the contributions of COPD severity.

The limitation of physical activity due to COPD can theoretically diminish a sexual function of patients. Sexuality is a topic that has rarely been studied in COPD patients and this item is evidently unrecognized by airway disease-specific questionnaires. As a more comprehensive instrument the 15D, contrary to other generic, including preference-based instruments, covers sexual functioning. Interestingly, in our study sexual functioning correlated significantly to respiratory symptoms. The correlation was at the same level as with mobility, breathing, usual activities, and vitality. The sexual quality of life was negatively affected in both genders, but men reported significantly worse sexual function (data not shown). One reason for that could be an erectile dysfunction reported in a study of outpatients with COPD varying with the disease severity [43].

Insufficient data have been published on the use of HRQoL instruments in clinical practice. The use of the HRQoL instruments in everyday clinical settings is limited by several factors. One obvious limitation is time: most questionnaires are time-consuming and therefore incompatible with everyday clinical practice. Respecting the clinician needs for short and fast, self-administered, valid and relevant instruments to measure HRQoL in COPD, the 15D tool could provide a means of eliciting information on areas which are otherwise difficult to identify and address during routine visits. Moreover, recent studies show clearly that COPD can no longer be regarded as a disease involving the lungs only. [37-39]. The 15D instrument captured the impact of both pulmonary and extra-pulmonary manifestations of the COPD patients and thus, offers an interesting and versatile choice not only to monitor COPD, but also assist clinician's decisions.

Furthermore, compared to respiratory specific instruments, a great advantage of this questionnaire is that, alike other generic preference-based instruments, it allows simultaneous cost-utility comparisons between different treatment interventions in COPD and even between other chronic conditions essential in public health care with limited resources.

## Conclusions

Scores of the corresponding questions and dimensions of AQ20 and 15D, respectively, and the summary scores of both instruments are comparable in terms of measuring HRQoL in COPD patients. The results of this comparative analysis support the convergent validity of 15D to measure the quality of life in COPD. COPD compromises the HRQoL broadly, as reflected by the generic instrument. Both questionnaires are simple and short, and could easily be used in clinical practice with high response and completion rates.

## Abbreviations

15D: fifteen dimensional; AQ20: Airway-specific questionnaire 20; COPD: chronic obstructive pulmonary disease; CRQ: Chronic Respiratory Disease Questionnaire; EQ-5D: EuroQol five-dimension questionnaire; GOLD: Global Initiative for Chronic Obstructive Lung Disease; HRQoL: health-related quality of life; HUI3: Health Utilities Index 3; MMRC: Modified Medical Research Council; NHP: Nottingham Health Profile; QWB: Quality of Well-Being Scale; R: correlation coefficient; SF-6D: short form six-dimension questionnaire; SF-36: Short Form 36-item Questionnaire; SGRQ: St. George's Respiratory Questionnaire; SIP: Sickness Impact Profile; VAS: visual analog scale

## Competing interests

Harri Sintonen is the developer of the 15D instrument. Apart from that the authors declare that they have no competing interests.

## Authors' contributions

WM collected the results, participated in the statistical analysis and drafted the manuscript. HK, HS, JP and VLK participated in the data collection, statistical analysis, and interpretation of the results and helped to draft the manuscript. MK and AL have participated in the study design and organised the execution of the study. TL designed and co-ordinated the clinical phase of the study, performed the statistical analysis, and supervised the manuscript preparation. All authors have read and approved the final manuscript.
